# Efficacy and Safety of Growth Hormone (GH) Therapy in Patients with SHOX Gene Variants

**DOI:** 10.3390/children12030325

**Published:** 2025-03-04

**Authors:** Giorgio Sodero, Federica Arzilli, Elena Malavolta, Marilea Lezzi, Fabio Comes, Antonietta Villirillo, Donato Rigante, Clelia Cipolla

**Affiliations:** 1 Department of Life Sciences and Public Health, Fondazione Policlinico Universitario A. Gemelli IRCCS, Institute of Pediatrics, Università Cattolica del Sacro Cuore, Largo Francesco Vito 1, 00168 Rome, Italy; 2 Pediatric Department, Perrino Hospital, 72100 Brindisi, Italy; 3 Pediatric Endocrinology Unit, Perrino Hospital, 72100 Brindisi, Italy; 4 Periodic Fever and Rare Diseases Research Centre, Università Cattolica Sacro Cuore, 00168 Rome, Italy

**Keywords:** growth hormone, growth hormone therapy, SHOX gene, pediatric endocrinology

## Abstract

Background: Among the potential indications for growth hormone (GH) therapy is the presence of mutations in the SHOX (short stature homeobox-containing) gene, located in the telomeric pseudotautosomal region (PAR1) on the short arm of both sex chromosomes. Despite general recommendations supporting GH therapy in these cases, there is a lack of comprehensive evidence specifically evaluating its efficacy and safety in this subgroup of pediatric patients. Aim: The objective of this scoping review was to evaluate the efficacy and safety of growth hormone therapy in patients with SHOX gene variants, providing a narrative synthesis of the included studies. Materials and Methods: This scoping review was conducted in accordance with the Preferred Reporting Items for Systematic Reviews and Meta-Analyses (PRISMA) extension for scoping reviews. We summarized information extracted from 22 articles identified by our search strategy. Currently, only one randomized clinical trial has analyzed the efficacy profile of GH in patients with SHOX mutations. Results: Growth hormone is a valuable therapeutic aid for these patients. However, its prescription in children with SHOX gene mutations should consider the specific characteristics of each patient, similar to the approach taken for patients with idiopathic growth hormone deficiency (GHD). Conclusion: Growth hormone therapy in patients with SHOX gene alterations appears to be both safe and effective. However, longitudinal prospective studies and targeted clinical trials are necessary to confirm these findings. Despite this, GH remains one of the preferred hormonal therapies for patients with short stature and confirmed SHOX gene mutations.

## 1. Introduction

Growth hormone deficiency (GHD) is one of the most common causes of short stature, defined as a height below two standard deviations for sex and age based on growth charts specific to the patient’s ethnicity; in children, the estimated prevalence ranges from one in 4000 to one in 10,000. Furthermore, it is estimated that approximately 1–2% of children with a height below two standard deviations from the mean may be affected by GHD [1].

Although growth hormone (GH) therapy is primarily used in patients with idiopathic GH deficiency, it is well established that its potential applications extend to various conditions [2]; these include idiopathic short stature (IIS) (though authorized only in specific regions worldwide), patients with hormonal deficiencies secondary to inflammatory or neoplastic damage to the hypothalamic–pituitary region [3], Turner syndrome [4], Noonan syndrome [5], and other clinical conditions such as chronic kidney disease [6]. Not all of these patients exhibit a confirmed hormonal deficiency based on laboratory tests; however, they display clinical evidence of short stature and reduced growth velocity sufficient to justify, under specific conditions, hormonal therapy [2,5]. This treatment may be continued until the closure of the growth plates or, in some cases, even into adulthood when a definitive hormonal deficiency is demonstrated through the execution of new dynamic stimulation tests for growth hormone, or when an organic impairment of the hypothalamic–pituitary axis is shown to justify the growth hormone deficiency [1].

In addition to the classic indications in patients with GHD, the use of GH has been validated in various concurrent conditions such as Turner syndrome [4] or Noonan syndrome [5], where, although there is no impairment of hormonal secretion, a significant benefit is achieved on auxological parameters [1]. Among the most recent findings, early initiation of GH therapy in patients with Prader–Willi syndrome has been shown to improve hypotonia, leading also to beneficial effects on early psychomotor development, weight, and BMI [7].

From an endocrinological perspective, the therapeutic applications of growth hormone are rapidly expanding, reflecting the evolving understanding of its systemic effects and broad clinical utility [1]. Growing scientific evidence underscores that GH is not only a critical factor in promoting growth but also plays a pivotal role in various other physiological processes. Its effects extend beyond linear growth recovery, encompassing significant improvements in glyco-metabolic profiles, muscle trophism, and cardiac contractility, all of which contribute to overall health and well-being [8,9].

In patients with organic growth hormone deficiency, such as those who are post-neoplastic or have undergone treatments like radiotherapy, the therapeutic benefits of GH are even more pronounced [8]. These patients often experience significant metabolic disturbances, including insulin resistance, altered lipid profiles, and muscle wasting. GH therapy has been shown to enhance insulin sensitivity and improve lipid metabolism, making it a crucial intervention in managing these metabolic abnormalities. Additionally, GH supports the recovery of muscle mass and strength. This is particularly important in pediatric patients, where the preservation of muscle trophism is vital for physical function, physical activity, and overall growth.

GH therapy also plays a significant role in improving cardiac function, blood pressure regulation, and various body composition parameters [9]. One of the key benefits of GH therapy is its ability to reduce fat mass, which in turn leads to a decrease in cardiovascular risk [1]. The impact of GH therapy on body composition and cardiovascular health is well documented in adult patients with GHD [9]; however, while these effects are also expected to be present in pediatric patients, the data in this population are less extensive.

Among the potential recent indications for GH therapy is the presence of variants in the SHOX (short stature homeobox-containing) gene, located in the telomeric pseudotautosomal region (PAR1) region on the short arm of both sex chromosomes, which escapes X-inactivation [10].

The SHOX gene encodes a homeodomain transcription factor involved in the regulation of growth; SHOX haploinsufficiency results in short stature that is responsive to GH therapy [2,11]. It is also possible that many children diagnosed with short stature initially classified as GHD are carriers of undetected inactivating mutations in this gene at the time of diagnosis [12]. Indeed, the spectrum of manifestations associated with SHOX mutations and/or haploinsufficiency is highly variable, ranging from completely asymptomatic patients (even in the absence of short stature) [13] to more severe presentations characterized by dysmorphic features and profound mesomelic skeletal dysplasia [14].

The clinical severity of SHOX haploinsufficiency can vary significantly, even among family members carrying the same SHOX gene mutation [15]. The phenotype may include Madelung deformity of the wrist, recognized as the hallmark feature of Léri–Weill dyschondrosteosis (LWS), a frequent painful condition, which restricts wrist mobility and is characterized by a lateral profile resembling an upturned fork [16]. It likely arises from impaired growth of the distal radial epiphysis, resulting in radial bowing, premature epiphyseal fusion, dorsal displacement of the ulna, and wedging of the carpal bones [17]. In general, it is the combination of clinical, anthropometric, and radiological characteristics of the child that can guide towards a diagnosis of SHOX deficiency. Therefore, the use of validated scores, such as the Rappold score, can assist in identifying patients who require further genetic investigation [18].

In the clinical suspicion of SHOX gene alteration, targeted genetic testing is recommended. If the diagnostic confirmation is obtained, conventional GH stimulation tests are not required [1,2,19]. However, radiographs of the hand/wrist should be performed, and, in selected cases, imaging techniques may be used to study the forearms and lower legs [20]. GH therapy is generally recommended for these patients, although there is no summary of evidence specifically assessing the efficacy and safety profile of GH in this category of children.

The objective of this current scoping review was to evaluate the efficacy and safety of growth hormone therapy in patients with SHOX gene variants, providing a narrative synthesis of the included studies.

## 2. Materials and Methods

### 2.1. Study Selection

**Figure 1:**
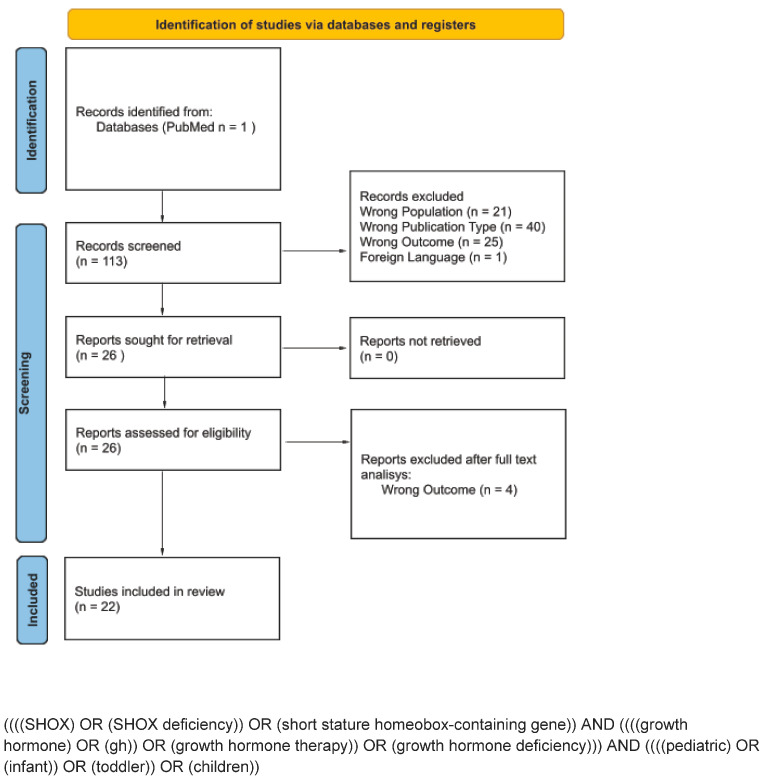
PRISMA flow diagram.

This scoping review was conducted in accordance with the Preferred Reporting Items for Systematic Reviews and Meta-Analyses (PRISMA) extension for scoping reviews [21]. The literature search strategy aimed to identify clinical studies evaluating the efficacy and safety of growth hormone (GH) therapy in patients with SHOX gene haploinsufficiency. The screening process, summarized in Figure 1, was carried out using multiple searches on PubMed.

The search string included the terms reported in the Supplementary Materials. The main question we aimed to address in this scoping review is the following: “What is known about the efficacy and safety of growth hormone therapy in patients with SHOX gene alterations?” We considered eligible for inclusion all original works published up to October 2024. Our scoping search followed the standardized SPIDER approach:

- Sample: pediatric patients with a confirmed diagnosis of SHOX gene alterations;
- Phenomenon of Interest: GH therapy in children with SHOX gene alterations;
- Design: randomized clinical trials, longitudinal studies, cross-sectional studies, and case reports;
- Evaluation: the efficacy and safety of GH therapy in patients with SHOX gene variants;
- Research Type: qualitative or quantitative methods.

Articles not fully available or not written in English were excluded during the preselection process. The screening procedure was independently performed by two authors of this manuscript (E.M. and F.A.) through an analysis of abstracts of all preselected articles, while a third author (G.S.) reviewed the full texts. Disagreements regarding study selection and data extraction were resolved by consensus among all authors.

From the initial studies selection (n = 113), we excluded studies that did not include patients with SHOX deficiency (n = 21), manuscripts written in languages other than English (n = 1), reviews of any type (n = 40), and studies with outcomes not aligned with the purpose of this review (n = 25).

A total of 26 publications were fully considered and included in this review. From the full-text screening performed by the authors, a total of 22 articles were analyzed and fully included in this scoping review; compared to the initial assessment, the full-text analysis revealed 4 manuscripts with outcomes that were not perfectly aligned with the focus of this scoping review. All included studies were analyzed according to the Grading of Recommendations, Assessment, Development, and Evaluations (GRADE) scale. Based on the authors’ considerations, none of the included manuscripts were deemed unsuitable for inclusion in the scoping review.

More details regarding our article screening and selection process are reported in the PRISMA diagram (Figure 1).

### 2.2. Data Extraction and Synthesis

The summarized information extracted from the 22 included articles are reported in Table S1.

The data extraction was performed by two authors (E.M. and F.A.) using a predefined Microsoft Office Excel form. This form included fields for references with DOI, authors, authors’ country, year of publication, type of study, primary aim and secondary aims (if reported), number of patients, genetic information (type of SHOX gene alterations), age, follow-up duration, height at the initiation of therapy and at the last endocrinological evaluation, any observed and/or reported side effects during GH treatment or treatment discontinuation, and main results.

The lead author also analyzed the conflicts of interest disclosed in the manuscripts to identify any potential biases in the interpretation of results. In cases of disagreement between the two authors, a third author (C.C.) was consulted. As previously stated, conflicting interpretations were resolved through collective discussion among all authors.

In addition to the preselected articles, we also reviewed the reference lists of all fully included manuscripts to identify any relevant studies not initially captured by our keyword combination.

Due to the heterogeneity of the publications analyzed, performing a meta-analysis of the results was not feasible.

## 3. Results

### 3.1. General Informations

A total of 22 manuscripts were included [22,23,24,25,26,27,28,29,30,31,32,33,34,35,36,37,38,39,40,41,42,43], published between 1999 (n = 1) and 2024 (n = 2). The majority of manuscripts were published in 2023 (n = 3), but there may be several ongoing prospective studies that could be published in the coming years. Only one randomized clinical trial (n = 1) [39] was identified through the screening, while many manuscripts (n = 8) were case reports regarding the therapeutic experience of a limited number of patients. The majority of the studies were conducted in Germany (n = 8), followed by Italy (n = 4). The total number of patients with SHOX alterations analyzed across the studies was 1355 children, with the largest study [37] including 521 patients. According to the GRADE scale, a total of seven studies were rated as high-quality, 12 studies as moderate-quality, and three studies as low-quality (Supplementary Materials).

### 3.2. GH Dosage Used and Follow-Up Duration

The average dosage of GH therapy across all studies was in line with that recommended by the guidelines, ranging from 0.03 mg/kg/day, while the highest dosage was 1.24 mg/m^2^ (or 0.048 mg/kg/day). The follow-up time varied across all manuscripts but lasted at least one year in all cases. The most commonly used average follow-up duration was 4 years, while the longest follow-up came from a retrospective study that analyzed data from 1995 to 2020. In 20/22 manuscripts, the primary objective was to evaluate the effectiveness of the therapy in patients with SHOX gene mutations, comparing them to patients with GHD or other syndromic conditions. In one study, efficacy data were derived from a larger cohort of patients receiving GH therapy with various underlying pathological conditions [30], with the main objective being the assessment of mortality in patients receiving hormonal therapy. A final study analyzed the relationships between GH treatment and carotid intima-media thickness [31], which is predictive of the development of atherosclerosis.

### 3.3. Benefit on Height and Growth Velocity

The benefit on growth velocity and height at the end of GH therapy was highlighted in nearly all of the analyzed manuscripts, although two manuscripts do not provide precise information regarding the height gain of the patients. The maximum increase in height observed in treated patients was +1.63 SD (mean value), in line with the efficacy information reported in the literature. No manuscript compared the different efficacy between pubertal and prepubertal patients, while one paper performed a comparison between patients with SHOX gene mutations and patients with Turner syndrome [42], showing a similar height benefit between the two conditions; these data are consistent with the genetic pathogenesis of Turner syndrome, as the short stature in this syndrome is caused by the reduced expression of the SHOX gene due to the absence of one X chromosome [44].

No study conducted a correlation analysis between the GH dose used and height increase, although a single study (not focused on height gain but on the occurrence of side effects) demonstrated that supraphysiological doses of GH do not result in a higher incidence of cardiovascular side effects compared to physiological doses [30].

### 3.4. Type of Mutation Reported

Due to the heterogeneity of the analyzed manuscripts and the variety of SHOX mutations, none of the studies attempted to compare the efficacy of the therapy in patients with different mutations. One study highlighted that patients with a deletion of the SHOX upstream enhancer region treated with growth hormone showed a better response to GH and a significantly greater benefit in terms of body proportions compared to patients with SHOX haploinsufficiency [22].

The phenotype–genotype correlation in patients with SHOX mutations, however, holds limited clinical significance, as the literature demonstrates that patients with identical mutations exhibit different growth parameters and expressivity [45]. Therefore, it is plausible that the response to growth hormone may also vary independently of the type of mutation [46].

### 3.5. Side Effects

The evaluation of the side effects of growth hormone treatment was not conducted in all the included manuscripts; in fact, only three papers provided information regarding the treatment’s side effects in patients, while four other manuscripts specified that no side effects occurred during the treatment. In the remaining papers, such information was not reported.

In all reported side effects, the complications were common among GH users [36], such as pain at the injection site, and no syndrome-specific complications related to the treatment were reported. However, this information is somewhat difficult to interpret, as the study of side effects was not the primary objective in any of the 22 studies analyzed. Therefore, it is possible that some data were not reported.

One study reported that one patient exhibited increased insulin resistance after approximately two years of therapy [43], a well-known side effect of growth hormone therapy that is also common in patients with idiopathic GHD. In the two studies not primarily focused on analyzing the height efficacy of GH therapy, it was emphasized that GH therapy does not appear to increase cardiovascular risk (assessed through carotid intima-media thickness) [31] or long-term mortality in patients with SHOX gene alterations [30].

### 3.6. Summary of Findings

Based on the information available in the literature, GH therapy in patients with SHOX gene mutations appears to be safe and effective, although there is significant variability in the follow-up time analyzed, the growth hormone dosage, and the clinical characteristics of the patients. None of the studies compared the efficacy between pubertal and prepubertal patients, although it is known that patients with hypothalamic–pituitary pubertal activation exhibit an initial acceleration in growth rate with early closure of the growth plates [47]. Growth hormone is a valuable therapeutic aid in these patients; however, its prescription in patients with SHOX gene mutations must take into account the specific characteristics of each patient, similarly to how it is performed in patients with idiopathic GHD.

## 4. Discussion

In this scoping review, we analyzed the efficacy and safety profile of growth hormone therapy in pediatric patients with SHOX gene haploinsufficiency. Our results demonstrated that growth hormone is both effective and well tolerated in this patient group, contributing to increased stature and improved growth velocity. Our findings align with the recommendations of major international scientific societies and, more generally, with the individual guidelines of various European and global countries [48,49], which increasingly include SHOX haploinsufficiency among the criteria for GH prescription with a dosage of 0.045–0.050 mg/kg/day, similar to that used in other syndromic conditions [4,5].

Short stature associated with SHOX deficiency can be suspected in patients with growth retardation and reduced growth velocity who exhibit distinctive bone features observed during bone age assessment [2,50]. These features include triangularization of the distal radial epiphysis, lucency of the ulnar border of the distal radius, enlarged diaphysis of the radius with bowing, short fourth and fifth metacarpals, pyramidalization of the carpal row, and convexity of the distal radial metaphysis [51]. While these indicators do not provide a definitive diagnosis, they serve as valuable markers suggesting the need for genetic investigations, particularly in patients with short stature and a normal response to dynamic testing for GH secretion [52]. The bone abnormalities observed in patients with short stature due to SHOX mutations have a multifactorial etiology, but they primarily depend on reduced expression of the protein encoded by the gene, which is expressed in the growth plates of growing bones and plays a role in modulating chondrocytes [53]. GH therapy, although it does not normalize the bone abnormalities in the carpal region, improves growth velocity and promotes statural development in patients, also enhancing the bone proportions of various body segments [22].

Based on the available literature, GH therapy in patients with SHOX gene mutations appears to be both safe and effective, although notable variability exists in the follow-up duration, GH dosage, and the clinical characteristics of the patients. This variability underscores the need for individualized treatment protocols tailored to each patient’s unique needs. Notably, none of the studies included in this scoping review compared the efficacy of GH therapy between pubertal and prepubertal patients, despite it being assumed that earlier initiation of GH therapy potentially has a greater impact on final height. This highlights the importance of considering pubertal status when determining the appropriate timing and dosage of GH therapy.

While GH therapy is a valuable treatment in patients with SHOX gene mutations, it is essential that its use be personalized, taking into account each patient’s specific clinical profile, much like the approach used for patients with idiopathic GHD. Future studies should focus on addressing the gaps in the current literature, including the comparison of treatment outcomes across different age groups and pubertal stages, to further optimize the management of these patients.

A recent scoping review [54] evaluated the efficacy profile of growth hormone therapy in various populations who are not growth hormone-deficient but may nonetheless benefit from treatment; comparing patients with and without growth hormone administration, the authors reported that patients with SHOX haploinsufficiency treated with rhGH therapy had an adult height +6.3 cm greater than that of untreated patients. The same review highlighted that the growth outcomes in these patients are comparable to those observed in individuals with Turner syndrome, with an improvement in adult height of up to +1 standard deviation compared to organic forms of GHD or untreated children [54,55]. Despite the internationally recognized benefits of this therapy, only one randomized clinical trial to date has prospectively assessed the efficacy of hormonal treatment in patients with SHOX deficiency. Blum et al. [39] conducted a study involving 52 prepubertal pediatric patients with a height below the third percentile for age and sex. Approximately half of the cohort (n = 27) received growth hormone (GH) therapy, while the remaining 25 patients served as untreated controls. The authors demonstrated that patients undergoing GH treatment exhibited a significantly greater first-year height velocity compared to the untreated control group (mean ± SE, 8.7 ± 0.3 vs. 5.2 ± 0.2 cm/year; *p* < 0.001), with results comparable to those observed in GH-treated individuals with Turner syndrome (8.9 ± 0.4 cm/year; *p* = 0.592). Additionally, GH-treated patients showed a significantly higher second-year height velocity (7.3 ± 0.2 vs. 5.4 ± 0.2 cm/year; *p* < 0.001), an improved second-year height standard deviation (SD) score (−2.1 ± 0.2 vs. −3.0 ± 0.2; *p* < 0.001), and a greater second-year height gain (16.4 ± 0.4 vs. 10.5 ± 0.4 cm; *p* < 0.001). In the study group, the incidence of side effects was not higher than that observed in patients typically treated with growth hormone, and the therapy was therefore deemed both effective and safe.

In the study that analyzed the largest number of patients with SHOX gene alterations [37] (n = 521, 314 female and 207 male), the authors highlighted an average improvement of +0.83 SD in patients treated with GH without significant side effects. The authors’ preliminary analyses also revealed that, similar to patients with idiopathic GHD, early initiation of therapy leads to a greater increase in final height without exposing the patient to a higher risk of side effects.

GH therapy should therefore be considered for this category of patients, despite the interindividual variability in growth response [56]. The treatment should be personalized in terms of dosage and duration, based on the auxological, clinical, and psychosocial characteristics of the child and their parents [57,58,59].

The use of growth hormone therapy in patients with SHOX gene alterations may in the future include new long-acting growth hormone formulations with weekly administration. Long-acting GH formulations have been shown to be safe and effective in children with growth hormone deficiency, demonstrating safety profiles comparable to daily GH, similar efficacy, and a significant improvement in therapy adherence [60]. However, these formulations are still under development, and in our country, they are authorized for a limited number of patients with fairly stringent indications [61]. Furthermore, differential data on efficacy and safety in different GHD etiologies are still unavailable. It is possible, however, that these long-acting formulations may eventually replace the traditional daily therapy, potentially becoming the preferred treatment in the future [62].

This manuscript has some limitations. It is a scoping review conducted by analyzing a single database, and it is possible that additional papers not indexed on PubMed could have been included if our search had been extended. Our keywords may also be imperfect, meaning that some relevant manuscripts may not have been perfectly captured by our search string and were therefore not included in the manuscript. Furthermore, due to the heterogeneity of the included manuscripts and the specific characteristics of the patients analyzed, we were unable to conduct a meta-analysis of results. Despite the limitations inherent in this scoping review, it offers a valuable synthesis of the current scientific evidence regarding the efficacy and safety of growth hormone therapy in pediatric patients with SHOX gene variants. This review consolidates a comprehensive understanding of the therapeutic outcomes, highlighting the clear benefits of GH treatment in this specific patient population. The findings presented here not only provide essential insights into the clinical application of GH therapy but also serve as a solid foundation for future systematic reviews and meta-analyses on the subject. These future studies will likely refine our understanding and help establish more precise treatment protocols for children with SHOX gene alterations. As GH therapy remains a cornerstone in the therapeutic management of these patients, its positive impact on growth parameters and overall development is well documented. This treatment continues to play a crucial role in enhancing the quality of life of children with SHOX gene variants, underscoring its undeniable clinical benefits. Additionally, ongoing research into new GH formulations, such as long-acting variants, promises to further improve patient adherence and outcomes, providing a compelling avenue for future clinical advancements in this field.

## 5. Conclusions

Growth hormone therapy in patients with SHOX gene variants is safe and effective. It improves growth velocity and target height in these patients, with no significant side effects beyond those already reported with the use of growth hormone in other types of patients, with or without GHD. Longitudinal prospective studies and targeted clinical trials are needed to confirm our findings. Nevertheless, regardless of this, GH is currently one of the preferred hormonal therapies for patients with short stature and confirmed genetic alterations of the SHOX gene.

## Supplementary Materials

The following supporting information can be downloaded at https://www.mdpi.com/article/10.3390/children12030325/s1: Table S1: Efficacy and safety of growth hormone (GH) therapy in patients with SHOX gene variants; the information regarding the assessment of the quality of the included articles is in accordance with the GRADE Working Group [63].
